# Reviewability and supportability: New complementary principles to empower research software practices^☆^

**DOI:** 10.1016/j.csbj.2024.10.034

**Published:** 2024-11-06

**Authors:** Haoling Zhang, Alberto Maillo, Sumeer Ahmad Khan, Xabier Martínez-de-Morentin, Robert Lehmann, David Gomez-Cabrero, Jesper Tegnér

**Affiliations:** a Biological and Environmental Science and Engineering Division, King Abdullah University of Science and Technology, 4700 Thuwal, Jeddah, 23955, Mecca, Saudi Arabia; b Computer, Electrical and Mathematical Sciences and Engineering Division, King Abdullah University of Science and Technology, 4700 Thuwal, Jeddah, 23955, Mecca, Saudi Arabia; c SDAIA-KAUST Center of Excellence in Data Science and Artificial Intelligence, 4700 Thuwal, Jeddah, 23952, Saudi Arabia; d Unit of Translational Bioinformatics, Navarrabiomed - Fundacion Miguel Servet, Universidad Pública de Navarra (UPNA), C. de Irunlarrea, 3, Pamplona, 31008, Spain; e Unit of Computational Medicine, Department of Medicine, Center for Molecular Medicine, Karolinska Institutet, Karolinska University Hospital, L8:05, Stockholm, SE-17176, Sweden; f Science for Life Laboratory, Tomtebodavagen 23A, Solna, SE-17165, Sweden

**Keywords:** Research reproducibility, Research software, Reviewability, Supportability, Development considerations, Peer review strategies, Deductive arguments

## Abstract

In today's scientific landscape, research software has evolved from being a supportive tool to becoming a fundamental driver of discovery, particularly in life sciences. Beyond its roots in software engineering, research software now plays a crucial role in facilitating efficient data analysis and enabling the exploration of complex natural phenomena. The advancements in simulations and modeling through research software have significantly accelerated the pace of scientific research while reducing associated costs. This growing reliance underscores the importance of software in ensuring reproducibility – a cornerstone of scientific rigor and trustworthiness. Although verifying reproducibility presents challenges, well-developed and openly accessible research software enhances transparency and aids in the early detection of errors. Although verifying reproducibility can be challenging, well-developed and accessible research software improves transparency and facilitates error detection. This mini-review examines the characteristics of research software and summarizes the key events that have shaped its development, alongside changes in requirements and guidelines. Moreover, we propose two additional principles – **reviewability** and **supportability** – complementing the widely accepted FAIR principles (Findability, Accessibility, Interoperability, and Reusability). These new principles aim to improve the efficiency and effectiveness of software evaluation during the peer review process. Through this review, we aim to assist scientists, especially those without extensive software development expertise, in understanding best practices for developing research software and the underlying motivations driving these practices.

## 1 Introduction

In modern research, research software, developed and employed to produce results for scientific publications [1], has become indispensable, transcending its role as mere auxiliary software tools to become central driving forces in scientific discovery. In life science research, from lab experiment design [2], [3], omics analysis [4], [5], molecular dynamics simulations [6], [7], advanced bioinformatics pipelines [8], [9], systems biology modeling of health and disease [10], [11], [12], [13], to molecular structure visualization, research software plays a pivotal role. Research software can empower scientists to design experiments precisely, analyze vast datasets efficiently, and uncover complex natural phenomena and underlying laws [14]. Meanwhile, advances in simulation from research software allow scientists to perform experiments and validations in silico, such as protein design [15] and antibiotic mining [16], significantly accelerating research progress and reducing costs.

The increasing reliance on research software has underscored its essential role in ensuring the reproducibility of scientific claims. Reproducibility is a gold standard for assessing the reliability of scientific claims [17]. However, as research works become increasingly complex, the challenge of verifying reproducibility also significantly intensifies. Hence, leveraging the self-replicating mechanism, research software's importance in supporting experimental results' reproducibility becomes increasingly clear. Properly developed and publicly available research software allows users to replicate every step of the research process, from data sampling to result analysis and figure plotting. This not only enhances the transparency of scientific claims but also provides opportunities to identify potential errors [18] and support secondary exploitation [19].

Because most research software is developed by scientists rather than software engineers, even when authors make it publicly available, ensuring that it fully supports scientific claims is often challenging, particularly for those without a software development background. Hereof, we begin by offering a property analysis of research software. Then, we examine the key incidents in the evolution of software development practices, along with the changes in requirements and guidelines. Finally, drawing from our experience in software development and software-related peer review, we outline the key qualities that make research software reviewable and detail the evidence chain required to demonstrate how research software can effectively support its associated scientific claims. Through this mini-review, we aim to help scientists in preparing better research software.

## 2 Property analysis of research software

A property analysis for research software is essential to foster a shared understanding of its unique characteristics compared with general-purpose software. Misalignment in the conceptual scope of research software may lead to less effective guidelines. This section offers the general definition and additional requirement consideration of research software to address this issue.

### 2.1 General definition

As the headword in the term “research software”, the term “software” has a wide-ranging definition [20], and theoretically, any process involving the interaction with computing resources can be defined as software. When the term “software” is qualified by the term “research”, its definition shifts accordingly. Currently, the widely accepted definition of research software refers to software used to generate, process, and/or analyze results intended for publications [1], emphasizing the distinction between research software and general-purpose software based on their specific usage. Additionally, a more detailed definition provided by Software and Source Codes College emphasizes the dual nature and flexibility of research software: it can both result from and support research, particularly concerning publications that occur before, during, or alongside its development. Research software can take various forms, such as a platform, middleware, workflow, library, or module/plugin integrated into another software. As a result, it may either interact within a broader ecosystem or function more independently.

### 2.2 Additional requirement consideration

In software engineering, the success of a software project largely depends on the effectiveness of requirements analysis [21]. Since the primary goal of research software is to support publications, it is essential to define its user base and specific purpose clearly. This understanding helps determine the components that should be included in the software and guides its proper development.

Before being formally presented to the academic community, publications typically undergo a peer review process [22]. This process plays a significant role in shaping the release workflow of research software, which often results in a user base (i.e., reviewers) that differs from that of general-purpose software during its initial stages. One key difference is that research software must meet additional requirements such as reproducibility and transparency to align with the standards set by the peer review process. Consequently, research software prioritizes scientific rigor over features like user-friendliness and broad applicability [14], setting it apart from general-purpose software products [23]. Moreover, the peer review process has time constraints and not all reviewers have extensive experience in code review. Thus, it becomes important to organize the material in a way that is easy for reviewers to follow, ensuring that it aligns with their thought processes and facilitates their understanding and assessment of the research software.

The role of research software in a publication determines which components should be disclosed to reviewers and the wider audience. The level of disclosure should align with the software's role in the research. It is neither reasonable nor realistic to expect all publications involving research software to meet the same or unique standards. The function of the research software can vary significantly between studies, from performing essential computational analyses to supporting more routine tasks. Conversely, even if research software is used only for basic tasks, such as figure plotting or data organization, failure to disclose it properly is still inappropriate. Even when its role seems minor, transparency is crucial. Thus, it is necessary to refine the element disclosure requirements for research software based on its specific purpose in publications.

## 3 Evolution of research software practices

As research software becomes more important in academic activities, its role is increasingly acknowledged in the academic community. This growing recognition often leads to heightened expectations (or, from another perspective, stricter requirements) and an influx of suggestions for software improvement. Tracing the historical evolution of these aspects provides a clearer understanding of the correct methods for developing research software and why doing so is essential. In this section, we examine the key events that have driven these changes, summarize the evolving expectations for research software in academic journals, and outline previous guidelines for software development.

### 3.1 Key incidents affecting software development

Every action has a purpose [24], considerations related to transparency and reproducibility did not simply appear out of nowhere. In this century, several key incidents, frequently marked by high-profile retractions or large-scale statistical results, have prompted the scientific community to consider better research software engineering practices continually.

The pivotal incident illustrating research software's influence on scientific claims' reproducibility was reported in 2006 [18]. A widespread retraction event occurred [25], [26], [27], [28], [29], [30] due to the erroneous inversion of electron-density maps of protein structures, attributed to a data-analysis program. This incident has been frequently cited as a case study emphasizing the need for improved research software engineering practices in modern research.

Unlike the unintentional program bug in this pivotal incident, most recent retractions have been driven by misconduct [66]. For the perspective of research software, misconduct often arises during the data usage phase (through the use of unverified [67], [68], [69] or fabricated [70], [71] data sources), the data handling process (using improper statistical methods [72], [73]), and the figure plotting process (where results are intentionally omitted [74], [75] or deliberately optimized [76], [77]). These instances of misconduct, coupled with overly optimistic results and the inadequate disclosure of the research software employed, enable these works to bypass the peer review process, leading to their eventual publication in prestigious journals.

### 3.2 Evolving expectations of academic journals

These key incidents have shaped academic journals' evolving expectations and requirements regarding the research software associated with publications. Tracking how these expectations and requirements evolve offers valuable insights into the standards and best practices necessary for developing research software effectively.

To trace the gradual requirement evolution for research software, we utilize editorials published in *Nature* and other Nature research journals as a lens, organized in Table 1, to illustrate the evolving expectations and requirements of the academic community regarding research software. Since the early 2000s, many editorials have highlighted the importance of research software. Initially, researchers were reluctant to share their published materials due to various reasons. Thus, a recurring topic was the encouragement of broader material sharing, including increasing the findability and accessibility of research software. As the significance of research software in publications grew and was influenced by the open-source community, the minimum disclosure requirements evolved. They gradually shifted from merely ensuring accessibility to mandating the provision of source code. Eventually, these requirements expanded to include data generated by source code, such as machine learning models, with heightened expectations for reusability.

**Table 1:** **Editorial history from *Nature* and other Nature research journals for research software issues.** We utilize a set of markers to categorize the editorial content:  recommend sharing the details;  recommend making software accessible;  recommend open-sourcing software;  recommend making software reusable;  recommend practical tools;  recommend correctly citing software;  require more precise descriptions;  require submitting a checklist with new reporting standards;  require making software accessible;  require open-sourcing software;  share successful applications or lessons learned;  release submitting and reviewing guidelines for software;  introduce a new section or initiate a pilot project for software.

Time	Journal name	Title	Marker(s)
2004-08	*Nature*	Share issues[31]	
2007-03	*Nature Methods*	Social software[32]	
2011-02	*Nature*	Devil in the details[33]	,
2013-04	*Nature Methods*	Enhancing reproducibility[34]	,
2013-10	*Nature Biotechnology*	In need of an upgrade[35]	,
2013-12	*Nature Genetics*	Credit for code[36]	,
2014-02	*Nature Methods*	Software with impact[37]	, ,
2014-09	*Nature*	The digital toolbox[38]	, ,
2014-10	*Nature*	Code share[39]	, ,
2014-10	*Nature Geoscience*	Towards transparency[40]	,
2015-04	*Nature Biotechnology*	Rebooting review[41]	, ,
2015-12	*Nature Methods*	Reviewing computational methods[42]	,
2017-05	*Nature Neuroscience*	Extending transparency to code[43]	,
2018-03	*Nature*	Does your code stand up to scrutiny?[44]	,
2018-08	*Nature Methods*	Easing the burden of code review[45]	,
2019-02	*Nature Physics*	A problem shared is a problem halved[46]	, ,
2019-02	*Nature Methods*	Giving software its due[47]	,
2019-05	*Nature Biotechnology*	Changing coding culture[48]	, ,
2019-08	*Nature Physics*	Correspondence on data[49]	,
2019-08	*Nature Machine Intelligence*	Sharing high expectations[50]	,
2020-12	*Nature Machine Intelligence*	Research, reuse, repeat[51]	,
2021-02	*Nature Computational Science*	Hail, software![52]	, ,
2021-07	*Nature Methods*	Computation and biology: a partnership[53]	,
2021-07	*Nature Computational Science*	But is the code (re)usable?[54]	, ,
2021-08	*Nature Human Behaviour*	Supporting computational reproducibility through code review[55]	,
2021-10	*Nature Methods*	Keeping checks on machine learning[56]	,
2021-10	*Nature Computational Science*	Moving towards reproducible machine learning[57]	,
2022-10	*Nature Machine Intelligence*	Revisiting code reusability[58]	, ,
2022-12	*Nature Computational Science*	Seamless sharing and peer review of code[59]	,
2023-05	*Nature Genetics*	Code deposition is unskippable[60]	,
2023-08	*Nature Astronomy*	Codes of honour[61]	,
2023-11	*Nature Computational Science*	Code sharing in the spotlight[62]	,
2023-12	*Nature Computational Science*	Formats for reporting primary research[63]	
2024-04	*Nature Machine Intelligence*	The rewards of reusable machine learning code[64]	,
2024-06	*Nature Ecology and Evolution*	Making the most of data sharing[65]	,

The editorial timelines for different journals in Table 1 also reveal the varying degrees of reliance on research software across disciplines, as well as how the unique characteristics of each discipline influence the accessibility of research software. Unlike other Nature research journals, *Nature Machine Intelligence* and *Nature Computational Science* have more comprehensive requirements for research software associated within their publications, reflecting the inseparability of research software from these fields. Additionally, in some disciplines, research software accessibility is often delayed due to hardware dependencies or policy requirements like embargo periods, which makes reviewability even more challenging. Despite the increasing availability of tools and platforms to aid in the accessibility of research software, identifying potential issues in research software may still be difficult within the peer review or short time frame of post-publication. To address these issues, *Nature* [38] and some Nature research journals [51], [61], [63] have introduced a specialized section to review the accessibility and reusability of their reported research software or analyze performances of various related research software for a field of research.

### 3.3 Discrete suggestions to systematic guidelines

Most research software is developed by scientists rather than software engineers, for whom the primary product is not the software itself but the resulting scientific insights [78]. This situation hinders allocating sufficient time and resources to develop research software within the broader scope of scientific activities [14]. Furthermore, since learning and using programming languages to implement specific functions or particular processes is merely the first step in the discipline of software engineering [79], many scientists may lack a clear understanding of how to develop appropriate research software.

To provide them with a basic understanding of developing research software in a relatively short period, experienced software engineers and scientists with extensive software development backgrounds have offered some simple suggestions, especially the continually updated “Ten Simple Rules” series by *PLOS Computational Biology* [80], [81], [82], [83], [84], [85], [86], [87], [88], [89], [90], [91], [92], [93].

Discrete suggestions may lack long-term applicability due to the continuous evolution of specific technologies and tools. Thus, several ambitious groups have begun exploring the potential feasibility of macro and high-level principles, with the FAIR principles being the most notable example [94]. These principles aim to enhance the findability, accessibility, interoperability, and reusability of scholarly data for humans and machines. After this, given that the most critical quality criterion for research software is functional correctness, the considerations for research software extend beyond the original principle scope, namely FAIR principles for Research Software (FAIR4RS) [95].

In recent years, the FAIR4RS principles have been continually refined [96]. Notably, the FAIR4RS working group, jointly convened by the Research Software Alliance, Future of Research Communications and e-Scholarship, and the Research Data Alliance, released a community-endorsed version of these principles in 2022 [97]:

• Findability: *Software, and its associated metadata, is easy for humans and machines to find*.
• Accessibility: *Software, and its metadata, is retrievable via standardized protocols*.
• Interoperability: *Software interoperates with other software by exchanging data and/or metadata, and/or through interaction via application programming interfaces, described through standards*.
• Reusability: *Software is both usable (can be executed) and reusable (can be understood, modified, built upon, or incorporated into other software)*.

Building on the FAIR4RS principles as well as integrating additional concepts from software engineering, more specific and systematic discussions are emerging. A key example is the progress of the Quality Research Software Task Force from the European Open Science Cloud (EOSC) Association. Their work covers a range of topics, including, but not limited to, the quality characteristics [98], [99] and lifecycle [100] of research software. One of this task force's primary goals is to identify qualitative and quantitative methodologies that can provide unbiased measurements of research software quality. Meanwhile, the efforts by the Software Sustainability Institute [101] and other groups are also contributing to the refinement of the systematic analysis of research software practices.

### 3.4 Practical infrastructures to support guidelines

To align research software with the expectations outlined in current guidelines, practical infrastructures offering essential services are indispensable. In 2023, the Quality Research Software Task Force undertook an evaluation of various scholarly infrastructures supporting research software [102], organizing them into four critical pillars:

• Archive: Long-term storage of research software and related materials for future access, through infrastructures like GitHub, GitLab, Zenodo, HAL, ArXiv, Figshare, e-cienciaDatos, RepOD, KU Leuven RDR, Digital CSIC, Dataverse, and InvenioRDM.
• Reference: Assigning unique identifiers to ensure accurate referencing and citation of research software, with infrastructures such asCodemeta, Bioschemas, swMATH, Research Software Directory, OpenAIRE, and GÉANT Software Catalogue.
• Describe: Providing detailed metadata and documentation to enhance accessibility and reusability of research software, through platforms including InvenioRDM, bio.tools, Bioschemas, Research Software Directory and Codemeta.
• Cite/Credit: Acknowledging research software in publications to give proper credit to creators, supported by Zenodo, Figshare, ArXiv, Digital CSIC, Dataverse, Dagstuhl Publishing, Episciences, European Mathematical Society Press, and Image Processing On Line.

In addition, there are platforms supporting reproducibility and traceability of research software like Snakemake, WorkflowHub, Code Ocean, NextFlow, and Docker. This preserves software quality and foster collaboration.

In life sciences, it is common to utilize language-specific repositories, such as CRAN for R or PyPI for Python, alongside domain-specific repositories like Bioconductor in R. Furthermore, Bioconda facilitates the distribution and installation of bioinformatics software packages across various platforms. For researchers with limited programming expertise, platforms like Galaxy offer a user-friendly, web-based interface for data analysis, enabling the creation, execution, and sharing of complex computational workflows.

These infrastructures collectively enhance the findability, accessibility, and sustainability of research software, while also promoting the principles of interoperability and reusability. Yet, the critical challenge remains in fostering further adoption of these tools and ensuring they effectively support the evolving needs of diverse research communities, particularly as the principles continue to be refined.

## 4 Shaping research software for reviewability

While the FAIR4RS guiding principles systematically enhance research software development from a software engineering perspective, they may not fully address the requirements unique to research software (as shown in Section 2). In particular, they lack adequate discussion from the perspective of the “research” component in “research software”. The primary purpose of providing supplementary materials alongside a manuscript is to strengthen the credibility of the scientific claims from multiple dimensions, ensuring greater transparency and reproducibility, research software is no exception. Nevertheless, the FAIR4RS principles do not explicitly specify which components of research software are crucial for achieving this objective. Hence, relying solely on these principles to develop research software may raise reasonable concerns. The worst-case scenario occurs when authors dedicate significant time to including an overwhelming amount of information in the research software that is not directly relevant to the scientific claims. This can cause reviewers to lose focus, making it difficult to conduct a thorough evaluation within the limited time available. Unfortunately, we have encountered this situation multiple times during the peer review.

Since reviewers are often the first research software users and may lack experience in code review, it's important to consider their needs during development. This helps authors and reviewers agree on the essential components and how the software should be presented. To address this, we introduce a new principle termed reviewability. It focuses on illustrating how easily reviewers can examine and evaluate research software. This section will explore this principle from two aspects: the dimensions of assessment and the specific components involved.

### 4.1 Initial impressions from reviewers

The initial impression of research software plays a critical role, as reviewers may form expectations about its overall structure before evaluating its detailed components. It can also preliminarily reveal the quality or maturity of evaluated research software [103]. Based on our experience, we propose two assessment dimensions for this initial impression: legibility (how research software can be presented) and completeness (what can be included in research software). Specifically, legibility is contingent upon the presence of the entry document, the organization of files, and whether the names of individual files or folders in archives can be associated with their respective roles. The file content level also includes details like naming conventions, code annotations, and comments. Completeness, conversely, is manifested in the existence of research software and its relevant materials, such as experimental configurations and results, automated tests, case studies, etc.

**Fig. 1:**
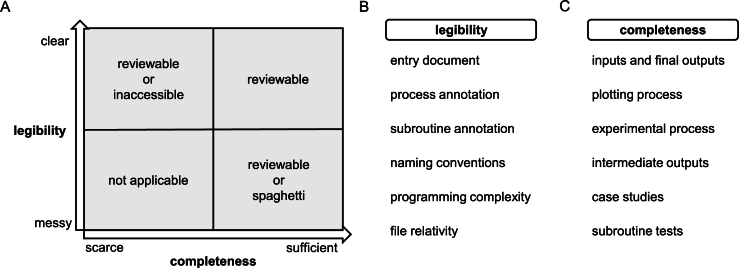
**Initial impressions and reference items.** (A) Two dimensions of initial impressions during the research software review. (B) and (C) Detailed reference items of each dimension.

As depicted in Fig. 1A, the combination of legibility and completeness can offer reviewers an initial perspective on the research software's reviewability during the peer review process. Based on the legibility and completeness, research software under peer review can be divided into three categories: reviewable, inaccessible, and “spaghetti” (a pejorative adjective describing codes and/or items that are challenging to maintain and/or lack structure [104]). There are normal forms from our peer review experience to illustrate inaccessible or what could be referred to as spaghetti research software. For sufficient but messy research software, it comprises dozens or even hundreds of files placed directly within an archive and lacks an entry document that indicates the organization or relationships among these files. On the contrary, for scarce but straightforward research software, only an entry document exists stating “updates will be coming soon” for a long time.

Beyond initial impressions, we reorganize early simple suggestions [80], [81], [82], [83], [84], [85], [86], [87], [88], [89], [90], [91], [92], [93] and form specific indicators for our proposed two assessment dimensions. This section outlines which components can be included in research software, rather than specifying those that must be included. For a more in-depth discussion of mandatory components, we will provide detailed explanations in Section 5.

### 4.2 Evaluation indicators for legibility

Legibility determines whether reviewers can complete the evaluation of research software within the limited time available, and it is the responsibility of the authors to guide this process effectively. As illustrated in Fig. 1B, legibility can be divided into six distinct evaluation indicators.

• Entry document: As the start point of research software, it is typically a Markdown file named README, which in some cases (e.g., the entire folder contains only one file) can have its contents integrated into an interactive document such as Jupyter Notebook. It contains, but is not limited to, the following information: a description of the software's objectives and functions, configuration and installation instructions, a file manifest detailing the contents of the archive, and operating instructions like execution steps;
• Process annotation: Sometimes, parts of the code may not be intuitive to readers. Hence, dividing the code according to functionality or purpose and providing inline or block comments can significantly enhance readability. It enables reviewers to focus more closely on the intricate details of the research software. Notably, before submitting research software as supplementary material for peer review, authors should ensure that annotations are in English rather than any other language to avoid reading difficulties;
• Subroutine annotation: Subroutines are presented by functions or methods. Functions are independent blocks of code designed to perform specific tasks, whereas methods are a type of function defined within a class and typically operate on instances of that class. Authors should provide annotations describing the functionality, parameters, and return values when implementing the subroutines. Subroutine annotations can include potential exceptions, usage examples, and performance considerations for more comprehensive documentation. Subroutine annotations have specific docstring formats. For example, four types of docstring format, Google, Sphinx/reStructuredText, NumPy, and Epytext, are recommended in Python;
• Naming conventions: They are not just about aesthetics; they reduce errors and make the code easier to understand. A common issue arises during the peer review process when authors use the same variable name in global and local environments. It can cause problems with the variable's life cycle, leading to errors in the results. Hence, adhering to naming conventions, like PEP 8 for Python, would be a best practice to establish a research software project;
• Programming complexity: It refers to the properties affecting internal interactions among entities (such as constants, variables, functions, and classes) [105]. In research software with complex entity interactions, it becomes challenging for reviewers or even the authors themselves to fully debug or review;
• File relativity: Items in the research software archive must be closely aligned with the relevant scientific claims. Including unrelated items, such as student transcripts, significantly burdens reviewers and negatively affects the credibility of the research software.

### 4.3 Evaluation indicators for completeness

As a prerequisite for the discussion in the subsequent subsection on the supportability of the research software, we need to focus on the completeness or valuable file composition of the archive shared by authors. Here, we temporarily disregard the differences between research software and their contexts and instead focus solely on all the considered items (and how to achieve them), as shown in Fig. 1C.

• Inputs and final outputs: These two types of data are formed as supplementary data. It allows reviewers to more accurately understand the information presented in figures from the manuscript and serves as a reference for future research. Supplementary data has been emphasized multiple times and is a mandatory submission requirement for most journals. Notably, clarifying the source of the supplementary data is equally important. A more pressing issue is that there are already cases where ChatGPT has been used to generate false data sets to support the unverified scientific claims [70], [71];
• Plotting process: Creating figures in the manuscript from the final outputs may not always be straightforward. Intentionally omitting specific values in final outputs or overfitting curves from final outputs can sometimes be difficult to detect [74], [75]. Thus, when figures are plotted using Matplotlib or other common libraries, providing the code that links the two materials can help reduce the possibility of improper figure processing and forgery. Executing the plotting process differs significantly from replicating the entire experiment. The associated code generally lacks complex environmental dependencies and can be repeated in a short amount of time.
• Experimental process: Under stricter peer review, reviewers may seek to understand the process of obtaining the final outputs, the experimental process. Due to the potential involvement of other software or statistical methods in the experimental process, authors might misuse or improperly use these tools because they are unfamiliar with their underlying principles. A concerning scenario arises when scientific claims become overly attractive, as they may result in profoundly negative societal impacts. For instance, vaccine hesitancy, identified by the World Health Organization as one of ten threats to global health, is exacerbated by the misuse of statistical methods [106]. In another recent case, a research article published in *Nature* was retracted on June 24, 2024, due to a methodological error falsely indicating a significant link between cancer and microorganisms [107], [108]. In a more typical scenario within bioinformatics and computational biology, the misuse of algorithm parameters or preprocessing steps during comparisons of different algorithms often results in the overestimation of the performance of certain algorithms [109], [110], [111]. This misuse can result in misleading evaluations of the algorithms' effectiveness. In a word, providing details of the experimental process could help address potential concerns from reviewers;
• Intermediate outputs: Beyond final outputs, intermediate outputs also play a crucial role in rigorous peer review and can be produced during the experimental process if properly configured. Their availability can further improve the transparency and reproducibility of research works [112]. Even though tools like Jupyter Notebook or cloud-based computational reproducibility platforms like Code Ocean make it easier to store intermediate outputs, authors may be hesitant to spend time on deployment and execution. Thus, reviewers will encounter interactive documents that haven't been executed, or they will see only environment logs as the outputs in the cloud-based computational reproducibility platform. Regardless, as an alternative, authors can consciously serialize intermediate outputs they obtain during their research process into files, which could then be delivered as supporting materials of scientific claims for reviewers to examine;
• Case studies: They can offer more precise descriptions to elucidate the functionalities of subroutines or the entire research software. Case studies focused on subroutines are instrumental in providing reviewers with a rapid deployment method to assess the correctness of these subroutines. Besides, end-to-end case studies encompassing the entire research software are crucial for demonstrating its practical application, thereby aiding reviewers in determining whether the research software meets its declared goals. Quick Start of Biopython [113] and Examples of Matplotlib [114] serve as the exemplary research software worth learning from in computation and visualization, respectively;
• Subroutine tests: The primary purpose is to demonstrate the implementation correctness of the subroutine objective(s). Given that thoroughly testing all possible inputs and verifying the correctness of all outputs is sometimes impractical, two commonly used methods are partitioning the input data into several equivalence classes and testing representatives from each class and testing the boundary values of the input data and the values near these boundaries [115]. When a subroutine's objectives are sufficiently complex, the aforementioned conventional methods may be difficult to implement or detect comprehensively. In such cases, employing alternative subroutine implementation for comparisons or utilizing the symmetry subroutine pairs, such as “encode-decode” or “serialize-deserialize”, for systematic testing can be effective if applicable. Adequate subroutine tests can help authors quickly identify potential errors and provide reviewers with valuable information regarding the correctness of research software implementation.

## 5 Supportability of research software

Although achieving all items mentioned in the valuable file composition can help significantly ease the peer review process and effectively support the scientific claims, several limiting factors, such as the degree of association between research software and scientific claims, software dependencies, and disclosure policies, should be considered. Here, we propose another new principle, supportability, which aims to clarify, in various contexts, which components are essential for research software to support the corresponding scientific claims adequately. Since overly strict and complex software submission guidelines may hinder the promotion and advocacy of best practices in research software development, it is worth discussing the minimal supportability of research software in different contexts to achieve balanced submission requirements for peer review.

### 5.1 Construction approach of evidence chain

Since research software places greater emphasis on supporting scientific claims than general-purpose software, it is crucial to focus on constructing an appropriate evidence chain within research software. This approach is no different from how evidence chains are constructed in scientific writing [116]. Even simpler, the supportability of research software can be effectively demonstrated by three sequential aspects: the inputs we have, the operations performed under specific configurations, and the outputs we obtain.

**Fig. 2:**
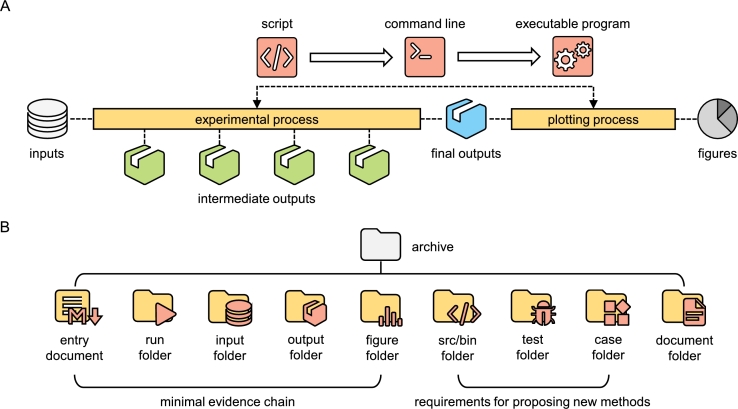
**Evidence chain built by research software and suggested file structure in the archive.** (A) The robust evidence chain can be specifically decomposed into six sequential parts. The order from script to executable program is the decreasing need for programming. (B) The archive of research software shared by authors can include up to nine components, some of which are optional depending on specific situations.

As shown in Fig. 2A, such an evidence chain can be specifically decomposed into six sequential parts: the inputs, the intermediate outputs, the final outputs, the experimental process, the plotting process, and the plotted figures. The experimental process and the plotting process both interact with the research software. Since these six parts are interdependent, they collectively form a robust evidence chain that sufficiently supports the corresponding scientific claims.

Notably, the term “figures” in Fig. 2A may not be fully equivalent to the figures presented in the manuscript. While we recognize that reorganizing different visual content to enhance the logical flow or information density of figures or altering the presentation focus to make the results more appealing to readers is generally reasonable, it is worthwhile to preserve the original visual content in the archive and compare it with the figures presented in the manuscript. This approach can reduce the risk of reviewers being misled by potential biases in the figures presented in the manuscript, allowing them to evaluate whether the authors' scientific claims are well-supported more accurately.

### 5.2 File structure for evidence chain

Drawing on our experience in developing and reviewing research software, we propose an efficient file structure with optional components for the archive that stores the research software and its relevant supporting materials, as shown in Fig. 2B. The entry document explains the archive, and in Section 4, we have detailed its necessary contents and the significance of its existence. Therefore, it is an essential file that must be present in the main directory of any research software archive. Based on the evidence chain, the run folder (encompassing scripts, command lines, and/or records of executable programs used in the experimental and plotting processes), the output folder (comprising intermediate and final outputs), and the figure folder (featuring raw figures derived from the plotting process and final outputs) should also be essential components. The importance of inputs depends on their source. In some cases, data may be generated during the experimental process, rendering the input folder optional in the archive. Thus, according to Fig. 2A, one essential file, three required folders, and one optional folder constitute the foundation (or minimal evidence chain) for supporting archive of research software.

For research works of establishing new algorithms, i.e., using custom-developed research software for experimental or plotting processes, an additional essential folder named “src/bin” should be included to store the relevant files. In most context, these files are source code. Nevertheless, due to commercial demands [117], [118], acceleration-driven goals [119], [120], [121], or necessary encapsulation under specific policies like security [122], [123] or sensitivity [124], this folder may also contain some pre-compiled binaries or consist entirely of pre-compiled binaries.

As required components, the custom-developed research software needs to undergo automated tests to verify their correctness and provide relevant case studies to demonstrate their use, detailed in Section 4. These two components can be placed in a “test” folder and a “case” folder, respectively, to ensure the clear file structure in the archive. It is worth highlighting, if the “src/bin” folder has lower readability (i.e., contains more pre-compiled binaries), it is advisable to include more tests and case studies to facilitate better evaluation by reviewers.

Reviewers may hesitate to thoroughly check all the details when the archive includes all the aforementioned components. To address this, some well-developed research software projects, such as Rosetta [119], have adopted a smart approach by creating a “document” folder. This optional folder can be linked to generate a website [87], allowing reviewers to quickly access sections of interest and encouraging broader participation in further development.

## 6 Interaction between concepts

**Fig. 3:**
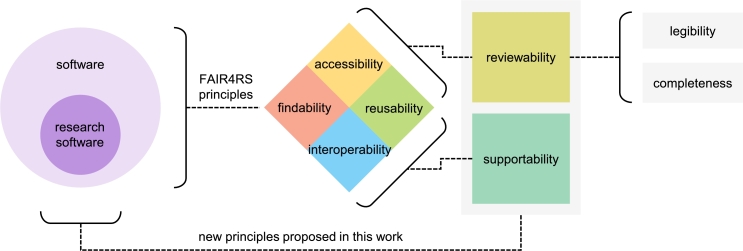
**Illustration of the interaction between mentioned concepts.** The concepts are “software”, “research software”, four components in FAIR4RS principles, “reviewability” (with its two dimensions “legibility” and “completeness”), and “supportability”.

Since this mini-review covers several concepts related to research software and introduces four new concepts, it is necessary to discuss the relationships between them, shown in Fig. 3.

As outlined in Section 2, we examine the distinction between software and research software. The latter is a subclass of the former, characterized by the qualifier “research”. Due to this qualifier, research software serves an additional specific objective (supporting the corresponding scientific claims) and caters to a special user base (i.e. reviewers) compared to general-purpose software.

To provide systematic practical guidance, the FAIR4RS principles, comprising findability, accessibility, interoperability, and reusability, were proposed from a software engineering perspective (as summarized in Section 3). Due to the lack of thorough discussion on the “research” aspect within research software in the FAIR4RS principles, they are more applicable to the broader concept of software than specifically to research software.

To more effectively address the “research” aspect within the concept of research software, we propose two new principles: “reviewability” and “supportability”. These new concepts are not isolated concepts. In essence, they offer a more detailed reinterpretation of the accessibility, interoperability, and reusability aspects of the FAIR4RS principles.

Findability is the cornerstone of reviewability and supportability, so discussing them without findability is pointless. Contrarily, the concepts of findability do not clearly overlap with those of reviewability and supportability.

For reviewability, the focus is on identifying the components that deserve attention from both authors and reviewers. Therefore, it overlaps with the vast majority of concepts related to accessibility. Nevertheless, the difference is that software metadata may not always be persistent regarding particular specific security and sensitive issues. Similarly, the download and access of the research software may be restricted to specific groups. Besides, reviewability encompasses aspects beyond accessibility. Reviewability includes relevant measures such as annotations and naming conventions to ensure reviewers can easily understand the research software shared by authors to achieve legibility, which is inherently related to reusability. However, since we discuss reviewability within the context of the peer review process, it does not include explicit requirements for shareability and portability. In some cases, the review process may not involve custom-developed research software. When the authors simply call different established software sequentially, modularizing the research software and providing communication protocols may not be applicable.

Since supportability is built on the foundation of reviewability, there is no direct correlation between supportability and accessibility. Unlike reviewability, which focuses on the presence of components, supportability emphasizes the interactions between these components from the reviewability perspective to ensure the research software can support its corresponding scientific claims. It explains how to achieve interoperability from a different viewpoint compared to reviewability. Besides, to provide a complete evidence chain, supportability must meet the requirement of replicating the same outputs as those in the scientific claims supported by research software, which is a key reusability aspect. Contrarily, supportability does not encompass the more complex scenarios covered by reusability [125], which include reusing the code with data other than the provided test data to obtain compatible output, applying the software to additional cases that reviewers believe it supports, and extending the software to enhance its functionality unless the authors explicitly disclose this capability.

## 7 Summary and outlook

This mini-review begins with a concise overview of the relevant background. Here we highlight the significance of research software in scientific activities and its role in ensuring research reproducibility through its self-replicating mechanism. Moreover, we conducted an in-depth analysis of the essential attributes of research software. We traced the evolution of research software practices through an examination of key incidents, shifting expectations in academic journals, and the development of best-practice guidelines.

Unlike previous works that further refines or implements the FAIR4RS guiding principles, our mini-review emphasizes the need to shift the focus from how to develop high-quality software to high-quality relevant research software which can support scientific claims. To this end, we introduce the concepts of reviewability and supportability from the reviewers' perspective, drawing on our peer-review experiences and case studies (especially retraction cases). We discuss how these proposed concepts can be effectively refined and implemented within the specific context of research software and examine their relationship to the definitions of the FAIR guiding principles.

Notably, we divide software codes in archives into three categories: experimental process, plotting process, and proposed algorithms. For most wet experiment-oriented work, developing new algorithms is typically not involved. For research focused on software development, it can be challenging to specify the exact form, whether it be source code or pre-compiled binaries, for various reasons. Regardless of the form, the proposed algorithms should produce the expected output based on the inputs mentioned in the paper. Unlike new algorithms, we emphasize the supporting role of experimental and plotting processes as the minimal evidence chain. To our knowledge, these process codes are crucial for the reliability of the scientific claims, and various reasons for not disclosing it are not applicable.

Although we provide a preliminary exploration for reviewability and supportability in this mini-review, the complexities stemming from disciplinary differences, commercial demands, acceleration imperatives, and the need for encapsulation under specific strategies highlight the critical need for broader involvement. Advancing these principles across different contexts requires flexible adaptation, rather than applying a one-size-fits-all approach with universal standards. Only by adjusting the scope of reviewability and supportability to suit different contexts can authors and reviewers reach the necessary consensus on whether research software effectively supports scientific claims during the peer review process. Therefore, expanding stakeholder engagement might be essential to advancing these principles across diverse contexts, which represents a fundamental commitment across all research fields involving research software to ensure the overarching goal of reproducibility.

Another promising catalyst is whether Large Language Models (LLMs) could help mitigate the difficulty in reaching this consensus. Aligned with the focus of authors, LLMs have already demonstrated significant potential in generating, optimizing, and/or debugging research software [126], [127], [128], [129], [130], [131]. Yet, a more thorough investigation is required to assess their efficiency in specialized academic domains beyond computer science (e.g., bulk and single-cell RNA-seq pipelines, detailed in the Supplementary Material). Furthermore, there is a notable absence of concrete metrics, such as those for measuring enhancements in code quality, to evaluate the performance of LLMs in these specific applications effectively. What is relevant to the interests of reviewers is whether, in the future, LLMs could be utilized to conduct a preliminary assessment of research software before formal peer review, generating a checklist for manual inspection. This could significantly reduce the workload for reviewers and enhance the reproducibility assessment before publication. Nevertheless, there are evident challenges in designing and training LLMs for this purpose. To date, no work has explicitly demonstrated that LLMs can effectively evaluate the interoperability and reusability of research software, let alone address principles that have not yet achieved widespread consensus (such as reviewability and supportability, as discussed in this work). Additionally, using LLMs in the peer review process underscores the need to implement robust privacy protection measures for the research software and its associated metadata under evaluation [132], [133]. Meanwhile, incorporating insufficiently validated LLMs into the peer review process could result in biased outputs [134], [135]. If reviewers rely too heavily on these automated systems without carefully examining the details, their judgment may be unintentionally influenced.

Overall, this mini-review outlines a crucial direction in how to foster reproducible research. It explores the properties and principles of research software and underscores the critical roles of reviewability and supportability in research software. Our ambition with this mini-review is to inspire continued development and application of the concepts presented, propelling the progress of reproducible research.

## CRediT authorship contribution statement

**Haoling Zhang:** Writing – original draft, Visualization, Resources, Methodology, Investigation, Conceptualization. **Alberto Maillo:** Writing – original draft, Validation, Software, Formal analysis, Data curation. **Sumeer Ahmad Khan:** Writing – review & editing, Validation, Resources. **Xabier Martínez-de-Morentin:** Writing – review & editing, Validation, Resources. **Robert Lehmann:** Writing – review & editing, Investigation. **David Gomez-Cabrero:** Writing – review & editing, Investigation. **Jesper Tegnér:** Writing – review & editing, Supervision, Project administration, Investigation, Funding acquisition, Conceptualization.

## Declaration of Competing Interest

The authors have no conflict of interest to declare.

## Appendix A Supplementary material

MMC Responses generated by four LLMs (Claude 3.5, ChatGPT-4, Unify AI, and Gemini) when asked to provide coding assistance for two case studies: the bulk RNA-seq case study and the scRNA-seq case study. Original and updated versions of the scripts are available on GitHub. MMC
