# Supplementary material for: Reviewability and supportability: New complementary principles to empower research software practices
Source: Comput Struct Biotechnol J. 2024 Nov 6;23:3989–98. doi: 10.1016/j.csbj.2024.10.034 (PMC11584522; doi:10.1016/j.csbj.2024.10.034)
Supplement: MMC — Responses generated by four LLMs (Claude 3.5, ChatGPT-4, Unify AI, and Gemini) when asked to provide coding assistance for two case studies: the bulk RNA-seq case study and the scRNA-seq case study. Original and updated versions of the scripts are available on GitHub. [file mmc1.pdf]

Supplementary material

Responses from the four LLMs, Claude 3.5, chatGPT, Unify AI, and Gemini, when asked to assist with the code of both case studies: Bulk RNA-seq Case Study and scRNA-seq Case Study. Original and updated scripts are accessible on GitHub: https://github.com/TranslationalBioinformaticsUnit/LLM_assistant_mini-review.

Bulk RNA-seq Case Study

Case Study: R script detailing the process from reading raw count tables per sample to normalization, including filtering steps.

Claude 3.5

Background: *I am a bioinformatician and have programmed this code, bulkRNA_casestudy.R, for my bulkRNA-seq analysis. Could you guide me by answering the following questions?*

Prompt 1: *Could you assist in writing good code following best practices?*

Response from Claude 3.5:

Here are the main improvements and best practices applied:

1. Consistent naming conventions: Used snake_case for variable names and function names.
2. Modularization: Created functions for reusable code blocks (e.g., *read_count_tables, filter_genes, get_ensembl_info*).
3. Error checking: Added *stopifnot()* statements to verify data integrity.
4. Improved readability: Added comments and organized code into logical sections.
5. File paths: Used *file.path()* for constructing file paths (more robust across operating systems).
6. Avoiding global variables: Passed necessary variables as function arguments.
7. Data visualization: Saved the boxplot to a PDF file for better reproducibility.
8. Data persistence: Saved the final results as both RData and CSV files.
9. Removed unnecessary variables and used more efficient data structures where possible.

This refactored code should be more maintainable, readable, and follow R best practices.

Prompt 2: *What are the best practices for developing an efficient code?*

Response from Claude 3.5:

Developing efficient code involves several best practices. Here are some key principles:

1. Optimize algorithms:
    - Choose appropriate data structures and algorithms for your specific problem.
    - Use efficient sorting and searching algorithms when dealing with large datasets.

2. Minimize redundant computations:
    - Cache results of expensive operations.
    - Use memoization for recursive functions.
    - Avoid unnecessary recalculations in loops.
3. Vectorize operations:
    - In R, use vectorized operations instead of loops when possible.
    - Utilize built-in functions optimized for performance.
4. Manage memory effectively:
    - Preallocate memory for large objects.
    - Remove large objects from memory when no longer needed (use *rm()* in R).
    - Use appropriate data types (e.g., integers instead of floating-point numbers when possible).
5. Leverage parallel processing:
    - Use multi-core processing for computationally intensive tasks.
    - Implement parallel algorithms when appropriate.
6. Profile and benchmark your code:
    - Use profiling tools to identify bottlenecks.
    - Benchmark different approaches to find the most efficient solution.
7. Optimize I/O operations:
    - Minimize disk reads and writes.
    - Use efficient file formats (e.g., binary formats for large datasets).
8. Use appropriate libraries and built-in functions:
    - Leverage optimized libraries for common operations.
    - Prefer built-in functions over custom implementations for standard operations.
9. Write clean, modular code:
    - Break complex tasks into smaller, manageable functions.
    - This allows for easier optimization and maintenance.
10. Avoid premature optimization:
    - Focus on writing correct code first, then optimize if necessary.
    - Profile your code to identify actual bottlenecks rather than guessing.
11. Consider lazy evaluation:
    - In languages that support it, use lazy evaluation to avoid unnecessary computations.
12. Use appropriate data types and structures:
    - Choose the right data structure for your needs (e.g., hash tables for fast lookups).
    - Use the most appropriate data type (e.g., int vs. float) to save memory and improve performance.
13. Optimize database queries:
    - If working with databases, optimize your queries and use indexing effectively.
14. Implement caching strategies:
    - Cache frequently accessed data to reduce computation time.
15. Consider using compiled languages for critical sections:
    - For extremely performance-critical parts, consider implementing them in a compiled language and interfacing with your main codebase.

Prompt 3: *What are the crucial checkpoints they should follow in the reviewing process?*

Response from Claude 3.5:

When reviewing bioinformatics code, especially for RNA-seq analysis, there are several crucial checkpoints to consider. Here are some key aspects to focus on during the review process:

1. Data Quality Control:
   - Check if raw data quality assessment (e.g., FastQC) was performed
   - Verify that low-quality reads and adapters were trimmed
2. Read Alignment:
   - Ensure the correct reference genome was used
   - Check alignment rates and distribution of reads across genomic features
3. Count Table Generation:
   - Verify that an appropriate method was used to generate count tables
   - Check for proper handling of multi-mapping reads
4. Filtering:
   - Ensure appropriate filtering of low-count genes
   - Verify that filtering criteria are justified and documented
5. Normalization:
   - Check that an appropriate normalization method was used (e.g., CQN in this case)
   - Verify that batch effects and other confounding factors were considered
6. Statistical Analysis:
   - Ensure the statistical model is appropriate for the experimental design
   - Check for proper handling of multiple testing correction
7. Reproducibility:
   - Verify that random seeds are set for reproducibility
   - Ensure all package versions are documented
8. Code Structure and Documentation:
   - Check for clear commenting and documentation
   - Verify that the code is modular and follows consistent styling
9. Data Integrity:
   - Ensure sample IDs are consistent across all steps
   - Check for unexpected data loss between steps
10. Visualization:
    - Verify that appropriate visualizations are included (e.g., PCA plots, heatmaps)
    - Ensure plots are clearly labeled and interpretable
11. Biological Interpretation:
    - Check if results are interpreted in a biologically meaningful context
    - Verify that appropriate databases or ontologies are used for functional analysis
12. Performance:
    - For large datasets, ensure the code is computationally efficient
    - Check for proper use of parallelization where appropriate
13. Error Handling:
    - Verify that the code includes appropriate error checking and handling
14. Version Control:

- Ensure the code is under version control (e.g., Git)
- Check for clear commit messages and logical version history

15. Data Management:
    - Verify that raw and processed data are stored appropriately
    - Ensure compliance with data protection regulations if working with sensitive data

ChatGPT

Background: *I am a bioinformatician and have programmed this code, bulkRNA_casestudy.R, for my bulkRNA-seq analysis. Could you guide me by answering the following questions?*

Prompt 1: *Could you assist in writing good code following best practices?*

Response from ChatGPT:

Here are some improvements and best practices for your R code:

1. Modular Structure: Break down the code into smaller, reusable functions.
2. Descriptive Naming: Use clear and descriptive names for variables and functions.
3. Documentation and Comments: Add comments to explain the purpose of each major section and function, including brief descriptions of function inputs and outputs.
4. Error Handling: Implement basic error checking to identify and address issues early.
5. Visualization: Include plots to assess quality control or show results.
6. Reproducibility: Ensure methods and results can be consistently replicated.

Summary of Changes:

- Modular Structure: The code has been broken down into functions (set_environment, read_count_tables, plot_boxplot, normalize_cqn, and main).
- Descriptive Naming: Function and variable names are more descriptive.
- Documentation and Comments: Each function has comments describing its purpose.
- Error Handling: Added stopifnot for checking conditions.
- Visualization: Added a boxplot for quality control before normalization.
- Reproducibility: Ensured methods are clear and functions can be reused

Prompt 2: *What are the best practices for developing an efficient code?*

Response from ChatGPT:

Developing efficient code is crucial for performance, maintainability, and scalability. Here are some best practices:

1. Modular Structure
    - Break Down Code: Divide your code into smaller, reusable functions or modules, each performing a specific task.
    - Encapsulation: Encapsulate related operations into classes or functions.
    - Single Responsibility Principle: Ensure each function or module has a single responsibility.
2. Descriptive Naming

  - Clear Names: Use clear, descriptive names for variables, functions, and classes to make the code self-explanatory.
  - Consistent Naming Conventions: Follow consistent naming conventions throughout the codebase.
3. Documentation and Comments
   - Function and Module Documentation: Document the purpose, inputs, outputs, and side effects of functions and modules.
   - Inline Comments: Add comments to explain complex logic or important sections of the code.
   - Comprehensive README: Include a README file with an overview of the project, setup instructions, usage examples, and dependencies.
4. Error Handling
   - Validation: Validate inputs and handle errors gracefully.
   - Logging: Implement logging to capture important events and errors.
   - Exception Handling: Use try-catch blocks or equivalent error-handling mechanisms to manage exceptions.
5. Efficiency and Performance
   - Algorithm Optimization: Use efficient algorithms and data structures.
   - Avoid Redundancy: Eliminate redundant calculations and data storage.
   - Parallel Processing: Leverage parallel processing or multi-threading where appropriate.
   - Profiling and Benchmarking: Profile and benchmark your code to identify and optimize performance bottlenecks.
6. Code Quality
   - Linting and Formatting: Use linters and formatters to maintain code quality and consistency.
   - Code Reviews: Conduct regular code reviews to ensure code quality and share knowledge.
   - Testing: Write unit tests, integration tests, and end-to-end tests to ensure code correctness.
7. Reproducibility
   - Version Control: Use version control systems like Git to track changes and collaborate.
   - Environment Management: Use tools like Docker, virtual environments, or package managers to ensure consistent environments.
   - Deterministic Outputs: Ensure that your code produces deterministic outputs given the same inputs.
8. Scalability and Maintainability
   - Scalable Architecture: Design your code and system architecture to scale with increasing data and user load.
   - Refactoring: Regularly refactor code to improve readability, reduce complexity, and adapt to changing requirements.
   - Dependency Management: Manage dependencies carefully to avoid conflicts and ensure compatibility.
9. Visualization and Logging
   - Data Visualization: Use data visualization to understand and communicate results.

    - Progress Logging: Log the progress of long-running operations for monitoring and debugging purposes.
10. Security
    - Secure Coding Practices: Follow secure coding practices to prevent vulnerabilities.
    - Access Controls: Implement proper access controls to protect sensitive data and resources.

Prompt 3: *What are the crucial checkpoints they should follow in the reviewing process?*

Response from ChatGPT:

In the code reviewing process, it's important to follow several crucial checkpoints to ensure the quality, functionality, and maintainability of the code. Here are the key checkpoints to consider:

1. Code Readability
    - Clarity: Ensure the code is easy to read and understand. Look for clear and descriptive naming conventions, consistent formatting, and appropriate use of comments.
    - Documentation: Check that functions, classes, and modules are properly documented with clear explanations of their purpose, inputs, and outputs.
2. Functionality
    - Correctness: Verify that the code performs the intended tasks correctly. Test the code with different inputs to ensure it behaves as expected.
    - Edge Cases: Consider edge cases and test how the code handles unexpected or extreme inputs.
3. Efficiency
    - Performance: Assess the performance of the code. Look for opportunities to optimize algorithms and data structures to improve efficiency.
    - Resource Usage: Check for unnecessary use of memory, CPU, or other resources. Ensure that the code is optimized for the given constraints.
4. Error Handling
    - Robustness: Ensure the code handles errors and exceptions gracefully. Look for appropriate use of try-catch blocks or equivalent error-handling mechanisms.
    - Validation: Verify that input validation is performed to prevent invalid data from causing errors.
5. Modularity and Reusability
    - Modular Structure: Ensure the code is broken down into small, focused functions or modules, each handling a specific task.
    - Reusability: Check if functions and modules are designed for reuse in other parts of the codebase or in future projects.
6. Code Quality
    - Linting and Formatting: Verify that the code adheres to the project's coding standards and style guidelines. Use linting tools to check for common issues.

- Consistency: Ensure consistency in naming conventions, code structure, and formatting throughout the codebase.

7. Testing
   - Unit Tests: Check that unit tests are written for individual functions and methods. Ensure the tests cover various scenarios, including edge cases.
   - Integration Tests: Verify that integration tests are in place to test how different parts of the code work together.
   - Test Coverage: Assess the test coverage to ensure that a significant portion of the code is tested.
8. Security
   - Secure Coding Practices: Check for common security vulnerabilities, such as SQL injection, cross-site scripting (XSS), and buffer overflows.
   - Access Controls: Ensure that sensitive data and resources are protected with proper access controls.
9. Maintainability
   - Refactoring: Look for areas where the code can be refactored to improve readability, reduce complexity, or eliminate duplication.
   - Dependency Management: Ensure that dependencies are properly managed and documented. Check for any potential conflicts or outdated libraries.
10. Version Control
    - Commit Quality: Review commit messages for clarity and relevance. Ensure that commits are logical, well-documented, and atomic (each commit represents a single change).
    - Branch Management: Verify that branches are used appropriately for feature development, bug fixes, and other tasks. Ensure that merges and pull requests are handled correctly.

Unify AI

Background: *I am a bioinformatician and have programmed this code, bulkRNA_casestudy.R, for my bulkRNA-seq analysis. Could you guide me by answering the following questions?*

Prompt 1: *Could you assist in writing good code following best practices?*

Response from UnifyAI:

Here are some improvements and best practices you can apply to your bulkRNA_casestudy.R script to make it more robust, readable, and efficient:

1. Use Descriptive Variable Names: Enhance readability by using more descriptive variable names.
2. Avoid Hardcoding: Reduce hardcoding, especially paths and parameters, to make the script more flexible.
3. Error Handling: Add error handling to manage potential issues during file reading or data processing.
4. Functions: Encapsulate repetitive logic into functions to make the code cleaner and more modular.
5. Use message(), warning(), and stop(): These functions are useful for debugging and error messages.

6. Code Comments and Structure: Improve comments to explain "why" something is done, not just "what" is done.

Key Changes:

1. Functions: Created read_and_process_counts to handle file reading and initial processing.
2. Error Handling: While not explicitly shown, consider adding checks after operations like read.table or getBM to ensure they complete successfully.
3. Modularity: Breaking down the script into functions like read_and_process_counts makes it easier to manage and debug.

Prompt 2: *What are the best practices for developing an efficient code?*

Response from UnifyAI:

Developing efficient code is crucial for creating software that is fast, resource-efficient, and scalable. Here are some best practices to consider when aiming to write efficient code:

1. **Understand the Requirements Clearly**: Before you start coding, make sure you fully understand the requirements. This helps in choosing the most appropriate data structures and algorithms from the start, which are crucial for efficiency.
2. **Choose the Right Data Structures and Algorithms**: The choice of data structures and algorithms has a significant impact on performance. Understand the time and space complexity of various options (Big O notation) and choose those that are most efficient for your use case.
3. **Write Clean and Readable Code**: Efficient code should not only perform well but also be easy to read, understand, and modify. Use meaningful variable and function names, keep functions focused on a single task, and organize your code logically.
4. **Avoid Unnecessary Computations**: Look for ways to reduce redundant calculations, such as caching results of expensive function calls when possible (memoization), and avoiding deep nested loops if simpler alternatives exist.
5. **Use Efficient Libraries and Frameworks**: Often, the most efficient way to implement a complex functionality is to use a well-optimized library or framework that specializes in that area. These are usually optimized and tested for performance and can save you a lot of time.
6. **Profile and Optimize**: Use profiling tools to identify bottlenecks in your code. Optimization should be based on profiling results rather than assumptions about what might be slow. Focus on optimizing the parts of the code that are used most frequently or that take up the most time.
7. **Consider Asynchronous Programming and Multithreading**: For I/O-bound and high-latency operations, consider using asynchronous programming to avoid blocking the execution. For CPU-bound tasks, multithreading or multiprocessing can be used to perform multiple operations in parallel, improving performance.
8. **Manage Memory Use Efficiently**: Avoid unnecessary memory consumption by managing resources properly. This includes deallocating resources that are no longer needed and understanding the memory usage patterns of your code.
9. **Minimize I/O Operations**: Input/output operations are often slow. Minimize the number of I/O operations, and try to perform them in bulk whenever possible. For instance, read and write large chunks of data at once instead of in many small operations.

10. **Use Compiler/Interpreter Optimizations**: Take advantage of compiler or interpreter options to optimize performance. Many compilers have options to optimize code for speed or memory usage.
11. **Keep Up with Advances**: Programming languages, compilers, and hardware are constantly evolving. Updates can include performance improvements, so keeping your environment and knowledge up to date can have significant benefits.
12. **Document and Review**: Document your optimizations and why they were necessary. Code reviews can also help identify potential inefficiencies and areas for improvement.

Prompt 3: *What are the crucial checkpoints they should follow in the reviewing process?*

Response from UnifyAI:

The code review process is a critical part of software development that ensures code quality, maintainability, and functionality. Here are some crucial checkpoints to follow during the reviewing process to maximize its effectiveness:

1. **Correctness and Functionality**:
   - **Does the code work?** Verify that the code correctly implements the intended functionality.
   - **Are there any bugs?** Look for logical errors, incorrect assumptions, edge cases, and potential bugs.
   - **Is there automated testing?** Check for the presence of unit tests and integration tests that cover new changes and important functionalities.
2. **Readability and Maintainability**:
   - **Is the code easy to understand?** Ensure that the code is clear and not overly complex.
   - **Are naming conventions followed?** Variables, functions, classes, and other entities should have clear, descriptive names.
   - **Is the code well-organized?** Good organization improves readability and maintainability.
   - **Is there sufficient commenting?** Comments should explain "why" something is done, especially for complex logic.
3. **Performance**:
   - **Is the code efficient?** Check for unnecessary computations, excessive memory use, and other inefficiencies.
   - **Can any part of the code be optimized without complicating the code?** Sometimes, small tweaks can significantly improve performance.
4. **Security**:
   - **Does the code introduce any security vulnerabilities?** Look for common security issues like SQL injections, cross-site scripting (XSS), and data leaks.
   - **Is sensitive data handled securely?** Ensure that data encryption, secure protocols, and other security best practices are used.
5. **Scalability**:
   - **Will the code scale well?** Consider how the code will perform under increased load.

- **Is the code modular?** Modular code can be more easily adapted or expanded.

6. **Compatibility and Integration**:
   - **Is the code compatible with existing systems?** Ensure that new code integrates smoothly with the existing codebase.
   - **Does the code adhere to API contracts and data formats?** Check for adherence to defined interfaces and expected data formats.
7. **Documentation**:
   - **Is the code adequately documented?** Documentation should be updated or added as necessary, particularly for APIs and complex logic.
   - **Are the changes well-documented in the version control system?** Commit messages should be clear and provide a good summary of the changes.
8. **Best Practices and Standards**:
   - **Does the code follow industry best practices?** This includes practices related to coding standards, design patterns, and architectural principles.
   - **Are there any deprecated or obsolete methods being used?** Code should use current best practices and technologies.
9. **Resource Management**:
   - **Are resources properly managed?** Look for potential resource leaks (e.g., database connections, file handles, network connections) and ensure they are properly handled.
10. **Team-Specific Criteria**:
    - **Does the code meet team-specific criteria and guidelines?** Each team might have its own set of practices, tools, or coding standards that need to be adhered to.

Gemini

Background: *I am a bioinformatician and have programmed this code, bulkRNA_casestudy.R, for my bulkRNA-seq analysis. Could you guide me by answering the following questions?*

Prompt 1: *Could you assist in writing good code following best practices?*

Response from Gemini:

Here are some suggestions for improvement based on best practices:

1. Readability:
   - Variable names: Consider more descriptive names like count_tables_path instead of count_tables_dir.
   - Comments: Add comments to explain specific sections, like data loading or filtering steps.
2. Organization:
   - Functions: Break down the code into functions for specific tasks like read_count_tables or filter_genes.
   - Data manipulation: Use functions from dplyr for filtering and manipulation instead of loops.
3. Efficiency:
   - Vectorization: Use vectorized operations instead of loops for better performance.

- Filtering: Consider using logical indexing for filtering instead of subsetting rows multiple times.

Prompt 2: *What are the best practices for developing an efficient code?*

Response from Gemini:

Here are some best practices for developing efficient code:

1. Focus on Readability:
    - Meaningful variable and function names: Use descriptive names that clearly explain their purpose. Avoid single-letter variables or overly cryptic abbreviations.
    - Comments: Add comments throughout your code to explain complex logic or non-obvious sections. This helps you and others understand the code in the future.
2. Prioritize Organization:
    - Modular functions: Break down your code into smaller, well-defined functions that perform specific tasks. This improves maintainability and reusability.
    - Logical code structure: Organize your code into clear sections with a logical flow. Use indentation consistently to enhance readability.
3. Leverage Built-in Functionality:
    - Utilize libraries and packages: Take advantage of existing libraries and packages that offer optimized functions for common tasks. This saves you time and ensures efficiency.
    - Vectorized operations: Use vectorized operations instead of loops whenever possible. Vectorized operations can handle entire arrays at once, leading to significant performance gains.
4. Optimize for Performance:
    - Profiling: Use profiling tools to identify bottlenecks in your code that slow it down. Focus optimization efforts on these areas.
    - Data structures: Choose appropriate data structures (e.g., dictionaries for fast lookups) depending on your needs.
    - Algorithmic efficiency: When writing your own algorithms, consider their time and space complexity. Opt for algorithms with lower complexity for large datasets.
5. Testing and Refinement:
    - Unit testing: Write unit tests to ensure individual functions work as expected. This helps catch errors early and prevents regressions during future modifications.
    - Benchmarking: Benchmark your code to compare its performance before and after optimizations. This helps quantify the impact of your changes.

Additional Tips:

- Minimize unnecessary operations: Avoid redundant calculations or data manipulations within your code.
- Utilize lazy evaluation: In languages like R, use lazy evaluation techniques (e.g., %>% operator) to chain operations efficiently.
- Explore alternative implementations: Research existing solutions or libraries that might offer more efficient ways to achieve the same functionality.

Prompt 3: *What are the crucial checkpoints they should follow in the reviewing process?*

Response from Gemini:

In the context of reviewing code for efficiency and best practices, here are some crucial checkpoints to follow:

1. Readability and Clarity:
    - Are variable and function names descriptive and easy to understand?
    - Is the code well-commented, explaining complex logic or non-obvious sections?
    - Is the code indentation consistent and easy to follow visually?
2. Organization and Structure:
    - Is the code broken down into well-defined and modular functions?
    - Does the code have a clear and logical flow?
    - Are there any unnecessary loops or redundant code blocks?
3. Efficiency:
    - Does the code utilize vectorized operations instead of loops whenever possible?
    - Are appropriate data structures chosen for the tasks at hand?
    - Could the chosen algorithms be optimized for better time or space complexity?
    - Is there any unnecessary data manipulation or redundant calculations?
4. Best Practices and Style:
    - Does the code adhere to established coding conventions and style guides for the chosen language?
    - Are there any opportunities to leverage existing libraries or packages for optimized functions?
    - Is the code properly formatted with consistent spacing and indentation?
5. Testing and Maintainability:
    - Are there unit tests in place to ensure individual functions work as expected?
    - Is the code well-documented for future reference and maintenance?
    - Could the code be easily modified or extended for future needs?

Additional Considerations:

- Purpose and Context: Review the code with its intended purpose and the nature of the data it handles in mind. Efficiency needs might differ depending on dataset size and processing requirements.
- Trade-offs: Sometimes, there might be trade-offs between efficiency and readability. Consider the balance between these factors depending on the project's specific needs.

.

scRNA-seq Case Study

Case Study: R script outlining the process from creating a Seurat object to identifying the most highly variable genes, including the normalization step.

Claude 3.5

Background: *I am a bioinformatician and have programmed this code, scRNA_casestudy.R, for my scRNA-seq analysis. Could you guide me by answering the following questions?*

Prompt 1: *Could you assist in writing good code following best practices?*

Response from Claude 3.5:

Key improvements and best practices applied:

1. Organized libraries at the top of the script.
2. Set a random seed for reproducibility.
3. Defined constants for frequently used values, improving readability and maintainability.
4. Used more descriptive variable names.
5. Added comments to explain each step of the analysis.
6. Improved plot generation and saving:
    - Combined related plots using patchwork.
    - Saved plots as PDF files for better quality and compatibility.
7. Streamlined the data processing pipeline.
8. Added error checking (implicitly through Seurat functions).
9. Saved the processed Seurat object for future use.
10. Generated a summary report with key information.
11. Followed consistent code styling (indentation, spacing).

This refactored code is more organized, readable, and follows R and bioinformatics best practices. It also includes steps to save results and generate reports, which are crucial for reproducibility and documentation.

Prompt 2: *What are the best practices for developing an efficient code?*

Response from Claude 3.5:

Developing efficient code is crucial in bioinformatics, especially when dealing with large datasets. Here are some best practices for writing efficient code:

1. Vectorization:
    - Use vectorized operations instead of loops when possible
    - Utilize functions like apply(), sapply(), lapply() in R
2. Memory management:
    - Remove large objects from memory when no longer needed (use rm())
    - Use data.table or dplyr for efficient data manipulation
3. Avoid redundant computations:
    - Cache results of expensive operations
    - Use memoization for recursive functions
4. Efficient data structures:

    - Choose appropriate data structures (e.g., data.frame vs. matrix)
    - Use sparse matrices for high-dimensional, sparse data
5. Parallelization:
    - Utilize parallel processing for computationally intensive tasks
    - Use packages like parallel, foreach, or BiocParallel in R
6. Profiling and benchmarking:
    - Use profiling tools to identify bottlenecks (e.g., Rprof() in R)
    - Benchmark different approaches to find the most efficient solution
7. Efficient I/O operations:
    - Minimize disk I/O by reading/writing data in chunks
    - Use efficient file formats (e.g., HDF5 for large datasets)
8. Algorithmic efficiency:
    - Choose efficient algorithms and data structures
    - Understand time and space complexity of your operations
9. Avoid unnecessary type conversions:
    - Be consistent with data types to avoid automatic type conversions
10. Use compiled code:
    - Integrate C++ code using Rcpp for performance-critical sections
11. Optimize database queries:
    - Use efficient SQL queries and indexing when working with databases
12. Lazy evaluation:
    - Take advantage of R's lazy evaluation when appropriate
13. Use appropriate libraries:
    - Utilize optimized libraries for common operations (e.g., data.table for data manipulation)
14. Code organization:
    - Modularize code for reusability and easier optimization
15. Memory-efficient data representation:
    - Use factor variables for categorical data in R
16. Avoid growing objects incrementally:
    - Pre-allocate memory for objects that will grow in size
17. Use appropriate precision:
    - Don't use higher precision than necessary (e.g., float vs. double)
18. Efficient string manipulation:
    - Use stringr or stringi packages for faster string operations in R
19. Optimize loops:
    - When loops are necessary, optimize by pre-allocating memory and minimizing function calls inside loops
20. Use efficient subsetting:
    - Understand and use efficient subsetting operations in R.

Prompt 3: *What are the crucial checkpoints they should follow in the reviewing process?*

Response from Claude 3.5:

When reviewing bioinformatics code, especially for single-cell RNA-seq analysis, there are several crucial checkpoints to consider. Here are key aspects to focus on during the review process:

1. Data Quality Control:
   - Verify that QC metrics (e.g., number of genes, UMIs, mitochondrial percentage) are calculated and visualized
   - Ensure appropriate thresholds for cell filtering are applied and justified
2. Data Preprocessing:
   - Check that normalization method is appropriate (e.g., log-normalization for Seurat)
   - Verify that variable feature selection is performed and parameters are justified
3. Dimensionality Reduction:
   - Ensure PCA is run on variable features
   - Check that an appropriate number of PCs are used for downstream analysis
4. Clustering:
   - Verify that a suitable clustering algorithm is used (e.g., Louvain or Leiden)
   - Ensure clustering resolution is appropriate and results are stable
5. Cell Type Annotation:
   - Check that a robust method for cell type identification is used
   - Verify that marker genes are appropriate for the tissue/organism studied
6. Differential Expression Analysis:
   - Ensure appropriate statistical tests are used
   - Check for proper multiple testing correction
7. Data Visualization:
   - Verify that informative plots are generated (e.g., UMAP, feature plots, violin plots)
   - Ensure plots are clearly labeled and interpretable
8. Code Structure and Documentation:
   - Check for clear commenting and documentation
   - Verify that the code is modular and follows consistent styling
9. Reproducibility:
   - Ensure random seeds are set for reproducibility
   - Verify that all package versions are documented
10. Data Integrity:
    - Check for unexpected data loss between analysis steps
    - Ensure sample IDs and metadata are consistently used
11. Batch Effect Correction:
    - If applicable, verify that batch effects are addressed appropriately
12. Trajectory Analysis:
    - If performed, ensure appropriate methods and visualizations are used
13. Functional Enrichment Analysis:
    - Verify that appropriate databases and statistical methods are used

14. Performance and Efficiency:
    - Check for computationally efficient code, especially for large datasets
    - Verify appropriate use of parallelization where necessary
15. Error Handling:
    - Ensure the code includes appropriate error checking and handling
16. Version Control:
    - Verify that the code is under version control (e.g., Git)
    - Check for clear commit messages and logical version history
17. Data Management:
    - Ensure raw and processed data are stored appropriately
    - Verify compliance with data protection regulations if working with sensitive data
18. Results Interpretation:
    - Check that biological interpretations are supported by the data
    - Ensure limitations of the analysis are acknowledged
19. Comparison to Existing Literature:
    - Verify that results are compared to existing knowledge where appropriate
20. Proper Use of Seurat Functions:
    - Ensure Seurat functions are used correctly and efficiently

By focusing on these checkpoints during the review process, you can help ensure the reliability, reproducibility, and robustness of the single-cell RNA-seq analysis. Remember to tailor these checkpoints to the specific requirements and context of your project.

ChatGPT

Background: *I am a bioinformatician and have programmed this code, scRNA_casestudy.R, for my scRNA-seq analysis. Could you guide me by answering the following questions?*

Prompt 1: *Could you assist in writing good code following best practices?*

Response from ChatGPT:

1. Added Section Comments:
    - Each major section (data loading, QC metrics visualization, normalization, feature identification, PCA analysis) is clearly marked with comments for better organization and readability.
2. Improved Variable Naming:
    - Consistent and descriptive variable names (pbmc.data, pbmc, plot1, plot2, top10) make the code more readable.
3. Enhanced Comments:
    - Additional comments explaining each code block and its purpose, making the code easier to understand and follow.
4. Visualization Emphasis:
    - Highlighted the importance of visualization (e.g., QC metrics, feature-feature relationships, PCA results) to aid in interpreting results and ensuring data quality.

5. Reproducibility Suggestion:
    - Mentioned setting a seed for random number generation to ensure reproducibility, a best practice for bioinformatics analysis..

Prompt 2: *What are the best practices for developing an efficient code?*

Response from ChatGPT:

Best Practices for Developing Efficient Code:

1. Modular Structure:
    - Break down the code into smaller functions to handle specific tasks such as data loading, QC metrics visualization, data normalization, feature identification, and PCA analysis.
2. Code Readability:
    - Use clear and consistent naming conventions for variables and functions.
    - Include comments to explain the purpose of each major section and function.
3. Error Handling:
    - Add checks to ensure the correct input data format.
    - Handle potential errors gracefully, for example, by verifying that the input files exist before attempting to read them.
4. Documentation:
    - Document the code with a clear explanation of each function's purpose, inputs, and outputs.
    - Include a README file with detailed usage instructions and dependencies.
5. Visualization:
    - Include visualizations to help interpret the results and assess the quality of the data at each step.
    - Ensure plots are clear and labeled appropriately.
6. Reproducibility:
    - Set a seed for any random number generation to ensure reproducibility.
    - Provide detailed information about the software environment and package versions used.

Prompt 3: *What are the crucial checkpoints they should follow in the reviewing process?*

Response from ChatGPT:

Crucial Checkpoints for Code Review:

1. Code Quality:
    - Ensure the code is clean, well-organized, and follows best practices for readability and maintainability.
2. Functionality:
    - Verify that the code performs the intended tasks correctly and handles edge cases appropriately.
3. Performance:
    - Assess the efficiency of the code, looking for opportunities to optimize performance and resource usage.

4. Testing:
    - Check that the code has been tested with a variety of inputs and that tests cover common use cases and edge cases.
5. Documentation:
    - Ensure the code is well-documented, with clear explanations of its purpose, inputs, outputs, and usage instructions.

Unify AI

Background: *I am a bioinformatician and have programmed this code, scRNA_casestudy.R, for my scRNA-seq analysis. Could you guide me by answering the following questions?*

Prompt 1: *Could you assist in writing good code following best practices?*

Response from Unify AI:

The key improvements made to your scRNA_casestudy.R script focus on enhancing readability, maintainability, efficiency, and adaptability. Here's a detailed breakdown of these improvements:

1. Improved Readability and Clarity:
    - Descriptive Variable Names: Changed variable names to be more descriptive (e.g., pbmc_data instead of pbmc.data). This helps in understanding what each variable represents without needing to trace its origin.
    - Structured and Commented Code: Organized the code into sections with comments explaining each part. This makes it easier for someone else (or future you) to understand what each block of code is intended to do.
2. Enhanced Maintainability:
    - Modular Code Blocks: By logically organizing the code into blocks that perform specific functions, it becomes easier to modify one part of the code without affecting others.
    - Use of Variables for Parameters: Using variables for parameters that might change (like data_dir) allows for easier updates. For instance, changing the data directory only requires updating one line in the script.
3. Increased Efficiency:
    - Avoided Redundant Operations: Removed unnecessary repeated function calls, such as the double call to NormalizeData. This not only saves computational resources but also avoids potential confusion about the script's logic.
    - Focused Data Processing: By subsetting the data early based on quality control metrics, subsequent operations are made more efficient as they operate on a reduced dataset.
4. Adaptability:
    - Parameterization: By defining paths and parameters at the beginning of the script, it becomes much easier to adapt the script to new data or different conditions without having to sift through the entire codebase to make changes.
5. Code Robustness:
    - Error Handling: While not explicitly shown in the code provided, adding error handling around file reads, data processing, and other critical operations can prevent the script from failing silently and make debugging easier if issues arise.

Prompt 2: *What are the best practices for developing an efficient code?*

Response from Unify AI:

Developing efficient code is crucial for creating software that is fast, resource-efficient, and scalable. Here are some best practices to consider when aiming to write efficient code:

1. **Understand the Requirements Clearly**: Before you start coding, make sure you fully understand the requirements. This helps in choosing the most appropriate data structures and algorithms from the start, which are crucial for efficiency.
2. **Choose the Right Data Structures and Algorithms**: The choice of data structures and algorithms has a significant impact on performance. Understand the time and space complexity of various options (Big O notation) and choose those that are most efficient for your use case.
3. **Write Clean and Readable Code**: Efficient code should not only perform well but also be easy to read, understand, and modify. Use meaningful variable and function names, keep functions focused on a single task, and organize your code logically.
4. **Avoid Unnecessary Computations**: Look for ways to reduce redundant calculations, such as caching results of expensive function calls when possible (memoization), and avoiding deep nested loops if simpler alternatives exist.
5. **Use Efficient Libraries and Frameworks**: Often, the most efficient way to implement a complex functionality is to use a well-optimized library or framework that specializes in that area. These are usually optimized and tested for performance and can save you a lot of time.
6. **Profile and Optimize**: Use profiling tools to identify bottlenecks in your code. Optimization should be based on profiling results rather than assumptions about what might be slow. Focus on optimizing the parts of the code that are used most frequently or that take up the most time.
7. **Consider Asynchronous Programming and Multithreading**: For I/O-bound and high-latency operations, consider using asynchronous programming to avoid blocking the execution. For CPU-bound tasks, multithreading or multiprocessing can be used to perform multiple operations in parallel, improving performance.
8. **Manage Memory Use Efficiently**: Avoid unnecessary memory consumption by managing resources properly. This includes deallocating resources that are no longer needed and understanding the memory usage patterns of your code.
9. **Minimize I/O Operations**: Input/output operations are often slow. Minimize the number of I/O operations, and try to perform them in bulk whenever possible. For instance, read and write large chunks of data at once instead of in many small operations.
10. **Use Compiler/Interpreter Optimizations**: Take advantage of compiler or interpreter options to optimize performance. Many compilers have options to optimize code for speed or memory usage.
11. **Keep Up with Advances**: Programming languages, compilers, and hardware are constantly evolving. Updates can include performance improvements, so keeping your environment and knowledge up to date can have significant benefits.
12. **Document and Review**: Document your optimizations and why they were necessary. Code reviews can also help identify potential inefficiencies and areas for improvement.

Prompt 3: What are the crucial checkpoints they should follow in the reviewing process?

Response from Unify AI:

1. Readability: I would check if the code is easy to read and understand. I would look for proper indentation, spacing, and naming conventions.
2. Comments: I would check if the code is well-documented with comments that explain the purpose of each section of the code.
3. Modularity: I would check if the code is broken down into smaller, manageable chunks. I would look for functions and pipes that make the code more modular and reusable.
4. Error handling: I would check if the code includes try-catch blocks to handle errors and exceptions.
5. Testing: I would check if the code includes tests to ensure it works as expected.
6. Code organization: I would check if the code is organized into logical sections, such as data loading, data preprocessing, and data analysis.
7. Code style: I would check if the code follows a consistent style, such as the tidyverse style guide.
8. Code reuse: I would check if the code reuses existing code whenever possible.
9. Code documentation: I would check if the code includes documentation that explains how to use it.
10. Code maintenance: I would check if the code is regularly reviewed and updated to ensure it remains accurate and efficient.

<u>Gemini</u>

Background: *I am a bioinformatician and have programmed this code, scRNA_casestudy.R, for my scRNA-seq analysis. Could you guide me by answering the following questions?*

Prompt 1: *Could you assist in writing good code following best practices?*

Response from Gemini:

1. Readability and Clarity:
   - Comments: Add comments to explain specific steps, particularly data loading and filtering choices.
   - Meaningful variable names: Consider renaming variables like pbmc.data to something more descriptive, like raw_expression_matrix.
2. Data Handling:
   - File paths: Consider storing file paths in a separate variable or object for better maintainability.
3. Code Organization:
   - Modular functions: Break down the code into functions for specific tasks like filter_low_quality_cells.
4. Efficiency:
   - Vectorized filtering: Use dplyr functions like filter() with logical vectors for efficient filtering instead of subsetting multiple times.

Prompt 2: *What are the best practices for developing an efficient code?*

Response from Gemini:

Here are some best practices for developing efficient code:

Focus on Readability:

- Meaningful variable and function names: Use descriptive names that clearly explain their purpose. Avoid single-letter variables or overly cryptic abbreviations.
- Comments: Add comments throughout your code to explain complex logic or non-obvious sections. This helps you and others understand the code in the future.

Prioritize Organization:

- Modular functions: Break down your code into smaller, well-defined functions that perform specific tasks. This improves maintainability and reusability.
- Logical code structure: Organize your code into clear sections with a logical flow. Use indentation consistently to enhance readability.

Leverage Built-in Functionality:

- Utilize libraries and packages: Take advantage of existing libraries and packages that offer optimized functions for common tasks. This saves you time and ensures efficiency.
- Vectorized operations: Use vectorized operations instead of loops whenever possible. Vectorized operations can handle entire arrays at once, leading to significant performance gains.

Optimize for Performance:

- Profiling: Use profiling tools to identify bottlenecks in your code that slow it down. Focus optimization efforts on these areas.
- Data structures: Choose appropriate data structures (e.g., dictionaries for fast lookups) depending on your needs.
- Algorithmic efficiency: When writing your own algorithms, consider their time and space complexity. Opt for algorithms with lower complexity for large datasets.

Testing and Refinement:

- Unit testing: Write unit tests to ensure individual functions work as expected. This helps catch errors early and prevents regressions during future modifications.
- Benchmarking: Benchmark your code to compare its performance before and after optimizations. This helps quantify the impact of your changes.

Prompt 3: *What are the crucial checkpoints they should follow in the reviewing process?*

Response from Gemini:

In the context of reviewing code for efficiency and best practices, here are some crucial checkpoints to follow, specific to scRNA-seq analysis:

1. Data Handling and Readability:
   - Clarity and Comments:
     - Are variable and function names descriptive (e.g., raw_expression_matrix instead of pbmc.data)?
     - Does the code include comments explaining data loading, filtering choices, and complex steps?
   - Data Loading:

    - Are file paths stored in a separate variable or object for better maintainability?
    - Could comments be added to clarify specific data loading parameters?

2. Code Organization and Structure:
    - Modular Functions:
        - Is the code broken down into well-defined functions for specific tasks (e.g., filter_low_quality_cells)?
        - Can further modularization improve readability and maintainability?
    - Logical Flow:
        - Does the code have a clear and logical flow from data loading to analysis steps?
        - Is the indentation consistent and visually appealing?
3. Efficiency and Best Practices:
    - Data Filtering:
        - Does the code utilize vectorized filtering with dplyr functions for efficiency?
        - Are there opportunities to avoid redundant filtering or calculations?
    - Libraries and Packages:
        - Does the code leverage appropriate Seurat functions and potentially other optimized packages for scRNA-seq analysis?
        - Could alternative functions or libraries offer better performance?
    - Algorithmic Choices:
        - Are the chosen algorithms (e.g., dimensionality reduction techniques) suitable for the data size and analysis goals?
        - Are there known optimizations for the chosen algorithms that could be applied?
4. Testing and Maintainability:
    - Reproducibility:
        - Is the code documented and clear enough for others to reproduce the analysis?
        - Are all necessary steps and data paths included?
    - Version Control:
        - Is the code version controlled using Git or a similar system for tracking changes and collaboration?
